# The chromosomal genome sequence of the sponge, *Corticium candelabrum *Schmidt, 1862 and its associated microbial metagenome sequences

**DOI:** 10.12688/wellcomeopenres.24923.1

**Published:** 2025-10-03

**Authors:** Manuel Maldonado, Lucia Pita, Dirk Erpenbeck, Ute Hentschel, Graeme Oatley, Elizabeth Sinclair, Eerik Aunin, Noah Gettle, Camilla Santos, Michael Paulini, Haoyu Niu, Victoria McKenna, Rebecca O’Brien, Wellcome Sanger Institute Tree of Life Management, Samples and Laboratory Team, Wellcome Sanger Institute Scientific Operations: Sequencing Operations, Wellcome Sanger Institute Tree of Life Core Informatics Team, EBI Aquatic Symbiosis Genomics Data Portal Team, Aquatic Symbiosis Genomics Project Leadership

**Affiliations:** 1 Department of Marine Ecology, Center for Advanced Studies of Blanes, Girona, Spain; 2 Instituto de Investigacions Marinas, Vigo, Galicia, Spain; 3 Department of Earth and Environmental Sciences & GeoBio-Center, Ludwig-Maximilians-Universität München, Munich, Germany; 4 GEOMAR Helmholtz Centre for Ocean Research Kiel, Kiel, Germany; 5 Tree of Life, Wellcome Sanger Institute, Hinxton, England, UK

**Keywords:** Corticium candelabrum, sponge, genome sequence, chromosomal, Homosclerophorida, microbial metagenome

## Abstract

We present a genome assembly from a specimen of *Corticium candelabrum* (sponge; Porifera; Homoscleromorpha; Homosclerophorida; Plakinidae). The genome sequence has a total length of 185.49 megabases. Most of the assembly (99.4%) is scaffolded into 22 chromosomal pseudomolecules. The mitochondrial genome has also been assembled and is 18.19 kilobases in length. Gene annotation of this assembly on Ensembl identified 26,198 protein-coding genes. The metagenome of the specimen was also assembled, and 53 binned bacterial genomes were identified, including 44 high-quality MAGs that were typical of high microbial abundance sponge and included, besides the phyla Chloroflexota (class Dehalococcoidia), Acidobacteriota (order Acidomicrobiales), Alpha- and Gammaproteobacteria, also representatives of several candidatus phyla (Candidatus Latescibacterota, Binatota, Poribacteria)

## Species taxonomy

Eukaryota; Opisthokonta; Metazoa; Porifera; Homoscleromorpha; Homosclerophorida; Plakinidae; *Corticium*; *Corticium candelabrum* Schmidt, 1862 (NCBI:txid121492)

## Background

*Corticium candelabrum* Schmidt, 1862 is a sponge species originally described from the sublittoral rocky bottoms of the Mediterranean region, where it is common. The species has also widely been reported from several Caribbean locations, the Atlantic Coast of Canada and Spain, the Indian Ocean, Indonesia, Papua New Guinea, New Zealand, and around Australia ( de Voogd *et al.*, 2024). The material used for this genome sequencing and the current species description are both derived from populations in the Western Mediterranean, the biogeographic region where the sponge was first described.

**Figure 1.**
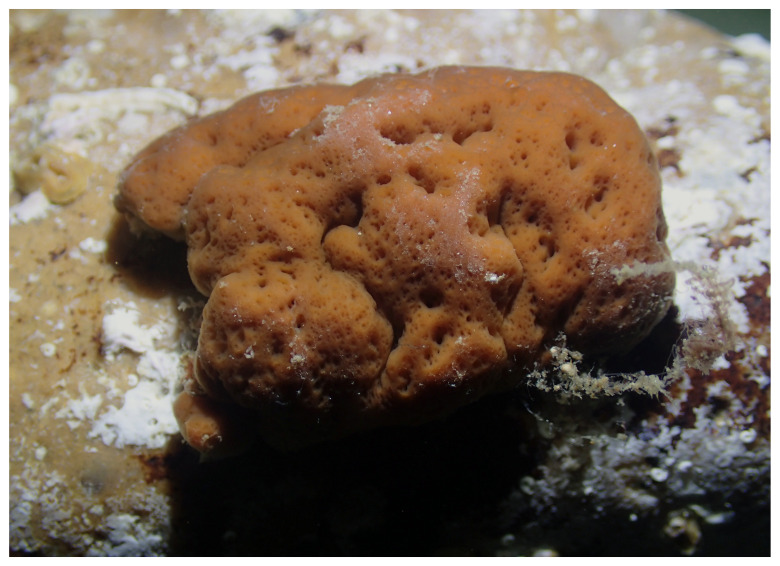
*Corticium candelabrum* specimen used for genome sequencing, photographed in the CEAB wet laboratory three hours after collection and immediately prior to tissue dissection. Underwater photography by Manuel Maldonado.

*Corticium candelabrum* is a light brown sponge that grows as lobate, cerebriform, plate-like individuals, reaching up to 10–12 cm in largest diameter and no more than 0.5 cm in thickness ( Figure 1). It has a smooth surface, with small osculi and ostia visible to the naked eye. The sponge typically inhabits rocky walls and overhangs and has no known predators other than the nudibranch *Platydoris argo* ( De Caralt *et al.*, 2007).

The species has notably helped to better understand the evolution of Porifera in several respects. This species was shown to have a basement membrane of type IV collagen beneath some epithelia, a condition typical of bilaterian metazoans and found only in sponges of the classes Homoscleromorpha and Calcarea ( Boute *et al.*, 1996; Wörheide *et al.*, 2012). The analysis of previous transcriptomes and the current genome has allowed to establish that Class Homoscleromorpha has independently evolved its own protein machineries for both transporting silicon and for making siliceous spicules, which are phylogenetically unrelated to those in the classes Demospongiae and Hexactinellida (Leria & Maldonado, under review; Maldonado *et al.*, under review; Shimizu *et al.*, 2024).

*Corticium candelabrum* is a hermaphroditic species that undergoes internal fertilisation and broods its embryos until the release of a cinctogastrula larvae ( Maldonado & Abdul Wahab, 2025; Maldonado & Riesgo, 2008). In Western Mediterranean populations, larval release occurs from mid-June to late-July, with dense populations producing an estimated half a million larvae per square metre annually ( Maldonado & Riesgo, 2008). The whitish larva is about 350 µm in length. It is entirely ciliated and swims for several days in the water column before settling and metamorphosing into a small juvenile sponge.

The microbiome of *Corticium candelabrum* is characteristic of high microbial abundance sponges, including ammonia-oxidising archaea and bacteria such as *Alpha- and Gammaproteobacteria*, *Actinobacteria*, *Candidatus* phylum Poribacteria, *Nitrospira*, and *Chloroflexi* ( Sharp *et al.*, 2007; Sipkema *et al.*, 2015). The process of vertical microbial transmission is well documented through electron microscopy studies ( De Caralt *et al.*, 2007; Maldonado, 2007; Riesgo *et al.*, 2007). Dense and diverse extracellular microbes are located particularly in subepithelial regions of the mesohyl and around oocytes and embryos ( Maldonado, 2007). The microbes surrounding the embryos migrate on their own and infiltrate the intercellular spaces that are formed between the blastomeres during the process of embryonic segmentation. As embryonic development progresses, a major cellular reorganisation occurs in a process analogous to gastrulation, wherein internal blastomeres migrate toward the periphery, leaving a central cavity, where all the microbes that had entered the embryo become finally enclosed.

The whole-genome sequencing of *C. candelabrum* provides a valuable tool to investigate the genomic underpinnings of its biology, ecology, and evolution. This genome is especially significant for understanding the evolutionary emergence of sponge classes, particularly the divergence between the Hexactinellida-Demospongiae and Homoscleromorpha-Calcarea clades. It also provides insights into the evolution of the siliceous skeleton of Porifera, as well as the origins of silicon biomineralization in Metazoa.

## Genome sequence report

### Sequencing data

The genome of a specimen of *Corticium candelabrum* ( Figure 1) was sequenced using Pacific Biosciences single-molecule HiFi long reads, generating 66.92 Gb from 6.19 million reads. Based on the estimated genome size, the sequencing data provided approximately 70 coverage of the genome. Chromosome conformation Hi-C data produced 142.68 Gb from 944.91 million reads. RNA sequencing data were also generated and are available in public sequence repositories. Table 1 summarises the specimen and sequencing information.

**Table 1.** Specimen and sequencing data for *Corticium candelabrum*.

Project information			
**Study title**	Corticium candelabrum		
**Umbrella BioProject**	PRJEB64714		
**Species**	*Corticium candelabrum*		
**BioSpecimen**	SAMEA9361900		
**NCBI taxonomy ID**	121492		
Specimen information			
**Technology**	**ToLID**	**BioSample accession**	**Organism part**
**PacBio long read sequencing**	ooCorCand1	SAMEA9361972	Somatic tissue
**Hi-C sequencing**	ooCorCand1	SAMEA9361954	Somatic tissue
**RNA sequencing**	ooCorCand1	SAMEA9361956	Somatic tissue
Sequencing information			
**Platform**	**Run accession**	**Read count**	**Base count (Gb)**
**Hi-C Illumina NovaSeq 6000**	ERR11814104	9.45e+08	142.68
**PacBio Sequel IIe**	ERR11809138	2.40e+06	28.7
**PacBio Sequel IIe**	ERR11809139	1.81e+06	17.65
**PacBio Sequel IIe**	ERR14749924	1.98e+06	20.57
**RNA Illumina NovaSeq 6000**	ERR11814103	3.65e+07	5.52
**RNA Illumina NovaSeq X**	ERR14986693	8.13e+07	12.28

### Assembly statistics

The primary haplotype was assembled, and contigs corresponding to an alternate haplotype were also deposited in INSDC databases. The assembly was improved by manual curation, which corrected 55 misjoins or missing joins and removed one haplotypic duplication. These interventions decreased the scaffold count by 9.38% and the scaffold N50 by 40.2%. The final assembly has a total length of 185.49 Mb in 115 scaffolds, with 277 gaps, and a scaffold N50 of 8.52 Mb ( Table 2).

**Table 2.** Genome assembly data for *Corticium candelabrum*.

Genome assembly	
Assembly name	ooCorCand1.1
Assembly accession	GCA_963422355.1
*Alternate haplotype accession*	*GCA_963422425.1*
Assembly level for primary assembly	chromosome
Span (Mb)	185.49
Number of contigs	392
Number of scaffolds	115
Longest scaffold (Mb)	16.58
Assembly metric	Measure
Contig N50 length	1.3 Mb
Scaffold N50 length	8.52 Mb
Consensus quality (QV)	Primary: 52.3; alternate: 52.4; combined 52.3
BUSCO *	C:79.9%[S:79.1%,D:0.7%],F:10.0%,M:10.2%,n:954
Percentage of assembly assigned to chromosomes	99.2%
Organelles	Mitochondrial genome: 18.19 kb
Genome annotation of assembly GCA_963422355.1 at Ensembl	
Number of protein-coding genes	26,198
Number of non-coding genes	413
Number of gene transcripts	40,526

* BUSCO scores based on the metazoa_odb10 BUSCO set using version 5.3.2. C = complete [S = single copy, D = duplicated], F = fragmented, M = missing, n = number of orthologues in comparison.

**Figure 2.**
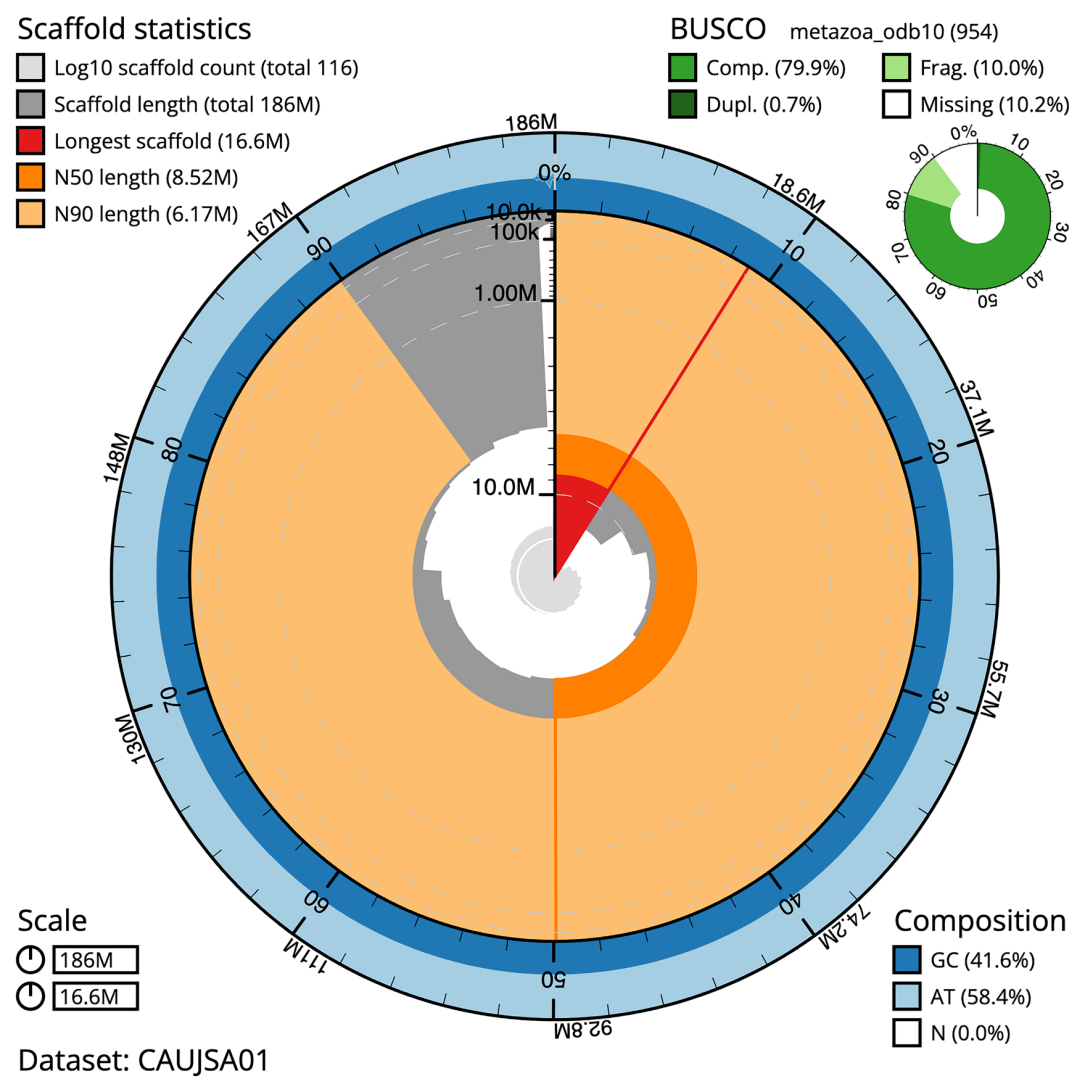
Genome assembly of *Corticium candelabrum*, ooCorCand1.1: metrics. The BlobToolKit snail plot provides an overview of assembly metrics and BUSCO gene completeness. The circumference represents the length of the whole genome sequence, and the main plot is divided into 1,000 bins around the circumference. The outermost blue tracks display the distribution of GC, AT, and N percentages across the bins. Scaffolds are arranged clockwise from longest to shortest and are depicted in dark grey. The longest scaffold is indicated by the red arc, and the deeper orange and pale orange arcs represent the N50 and N90 lengths. A light grey spiral at the centre shows the cumulative scaffold count on a logarithmic scale. A summary of complete, fragmented, duplicated, and missing BUSCO genes in the metazoa_odb10 set is presented at the top right. An interactive version of this figure is available at https://blobtoolkit.genomehubs.org/view/Corticium%20candelabrum/dataset/CAUJSA01/snail.

**Figure 3.**
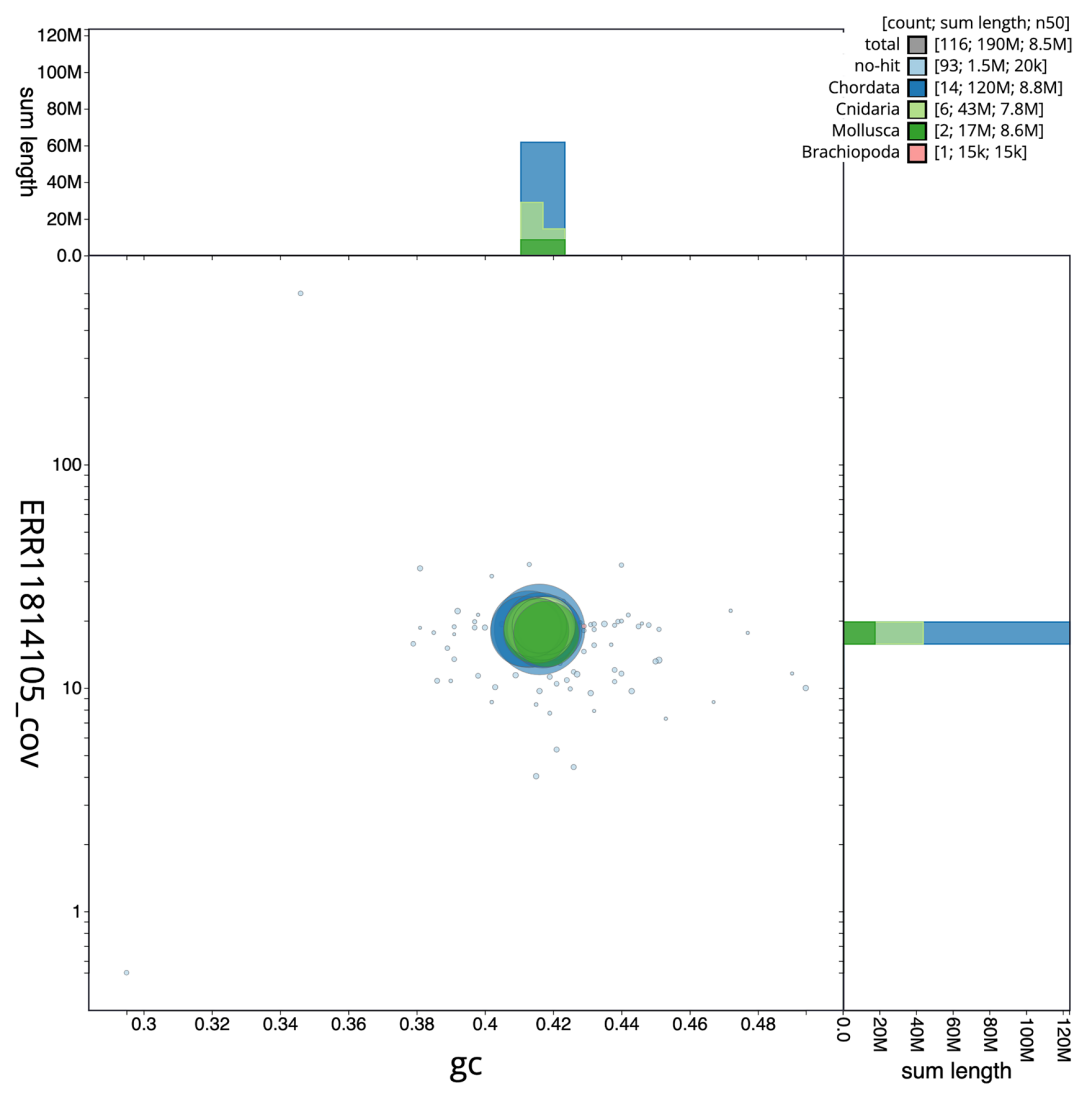
Genome assembly of *Corticium candelabrum*, ooCorCand1.1: BlobToolKit GC-coverage plot. Blob plot showing sequence coverage (vertical axis) and GC content (horizontal axis). The circles represent scaffolds, with the size proportional to scaffold length and the colour representing phylum membership. The histograms along the axes display the total length of sequences distributed across different levels of coverage and GC content. An interactive version of this figure is available at https://blobtoolkit.genomehubs.org/view/Corticium%20candelabrum/dataset/CAUJSA01/blob.

**Figure 4.**
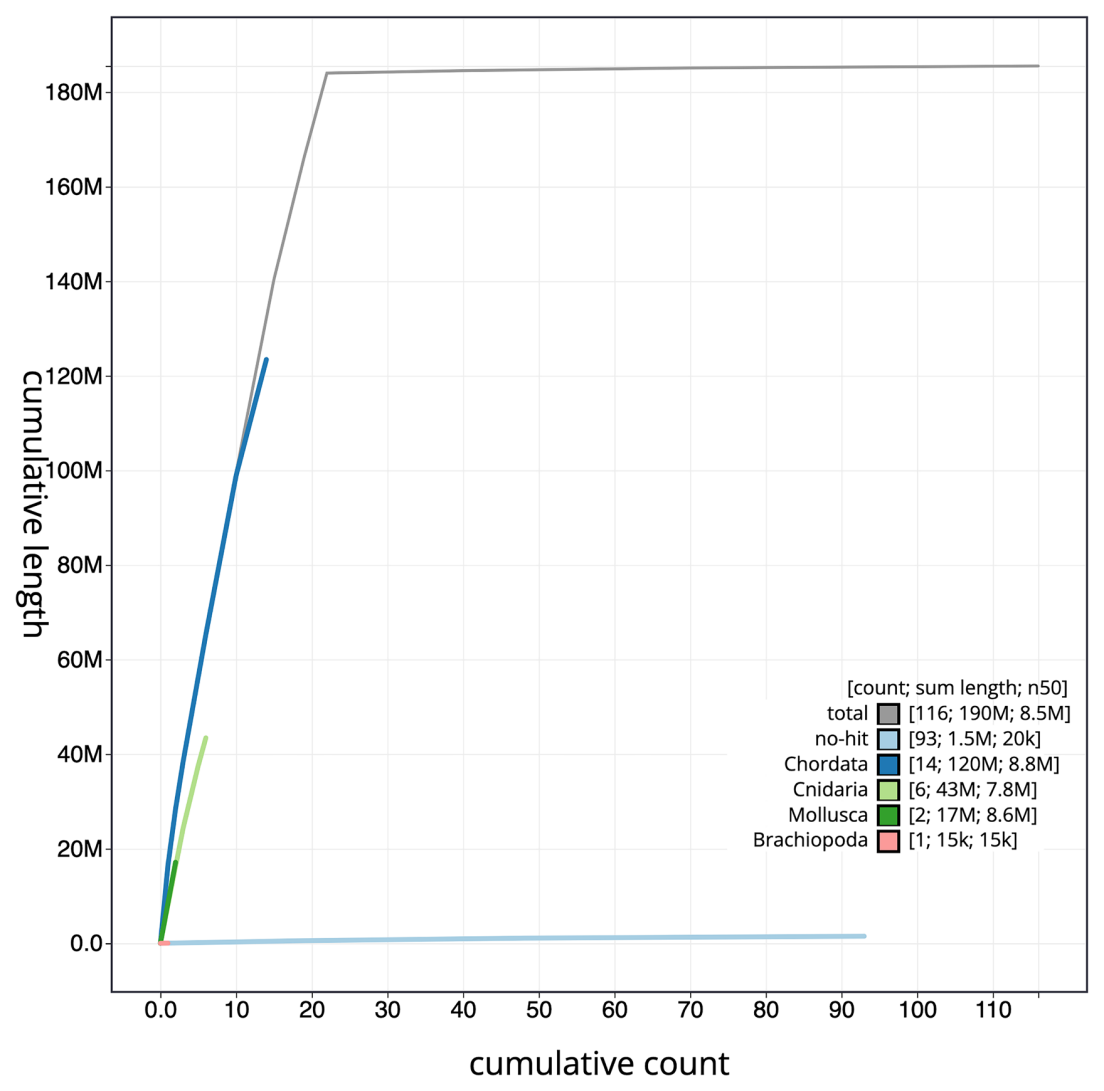
Genome assembly of *Corticium candelabrum,* ooCorCand1.1: BlobToolKit cumulative sequence plot. The grey line shows cumulative length for all scaffolds. Coloured lines show cumulative lengths of scaffolds assigned to each phylum using the buscogenes taxrule. An interactive version of this figure is available at https://blobtoolkit.genomehubs.org/view/Corticium%20candelabrum/dataset/CAUJSA01/cumulative.

The snail plot in Figure 2 provides a summary of the assembly statistics, indicating the distribution of scaffold lengths and other assembly metrics. Figure 3 shows the distribution of scaffolds by GC proportion and coverage. Figure 4 presents a cumulative assembly plot, with separate curves representing different scaffold subsets assigned to various phyla, illustrating the completeness of the assembly.

**Figure 5.**
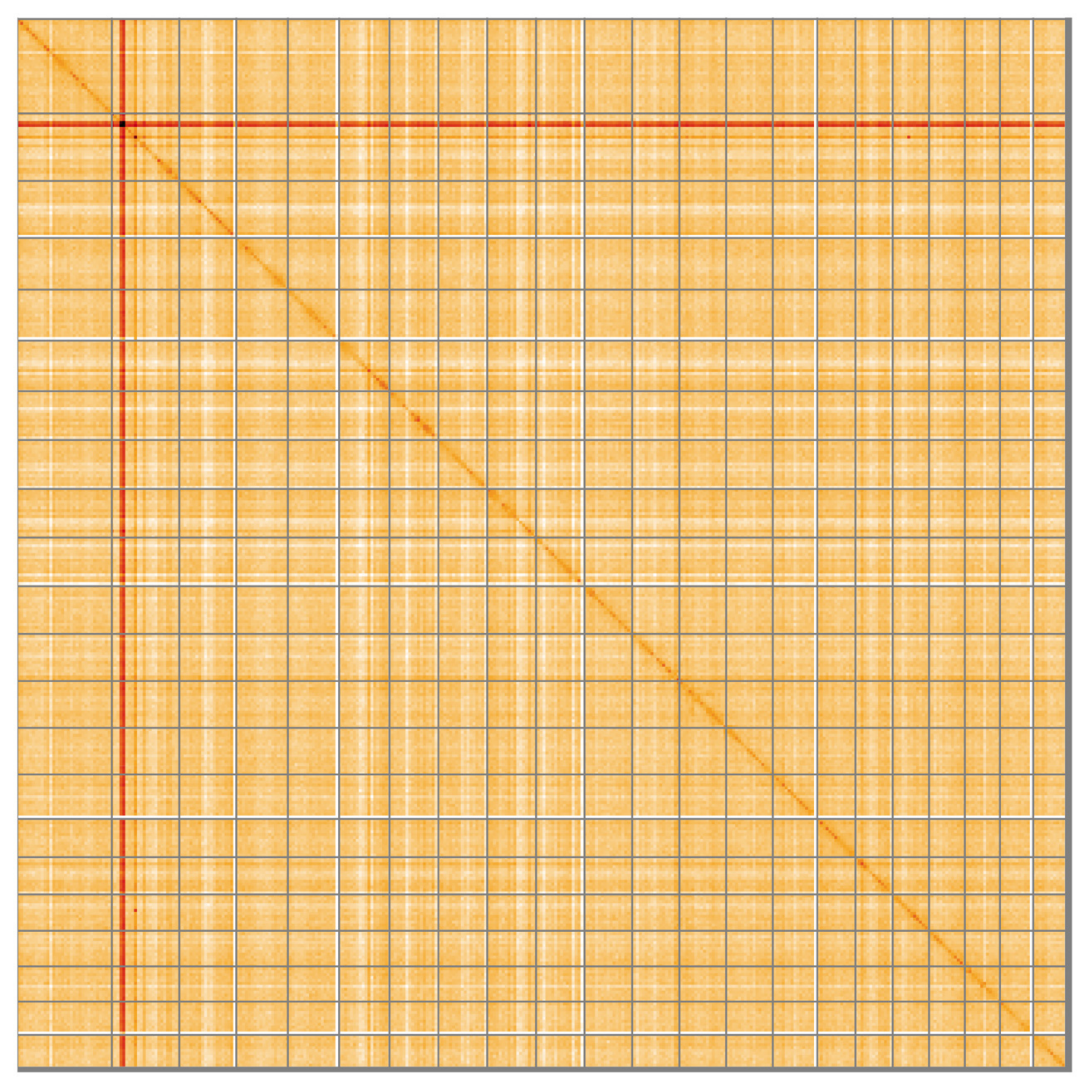
Genome assembly of *Corticium candelabrum*: Hi-C contact map of the ooCorCand1.1 assembly, visualised using HiGlass. Chromosomes are shown in order of size from left to right and top to bottom. An interactive version of this figure is available at https://genome-note-higlass.tol.sanger.ac.uk/l/?d=WjDYLGxASlOVoIXW1QIYRw.

Most of the assembly sequence (99.2%) was assigned to 22 chromosomal-level scaffolds. These chromosome-level scaffolds, confirmed by Hi-C data, are named according to size ( Figure 5; Table 3).

**Table 3.** Chromosomal pseudomolecules in the genome assembly of *Corticium candelabrum*, ooCorCand1.

INSDC accession	Name	Length (Mb)	GC%
OY729827.1	1	16.58	41.5
OY729828.1	2	11.83	41.5
OY729829.1	3	10.01	41
OY729830.1	4	9.05	42
OY729831.1	5	8.98	41.5
OY729832.1	6	8.85	41.5
OY729833.1	7	8.55	41.5
OY729834.1	8	8.58	42
OY729835.1	9	8.57	41.5
OY729836.1	10	8.52	41.5
OY729837.1	11	8.36	41.5
OY729838.1	12	8.22	42
OY729839.1	13	8.28	41.5
OY729840.1	14	8.17	41.5
OY729841.1	15	7.82	41.5
OY729842.1	16	6.7	41.5
OY729843.1	17	6.54	41.5
OY729844.1	18	6.37	42
OY729845.1	19	6.26	42
OY729846.1	20	6.17	42
OY729847.1	21	5.85	41.5
OY729848.1	22	5.74	41.5
OY729849.1	MT	0.02	34.5

The mitochondrial genome was also assembled. This sequence is included as a contig in the multifasta file of the genome submission and as a standalone record in GenBank.

### Assembly quality metrics

The combined primary and alternate assemblies achieve an estimated QV of 52.3. BUSCO v. 5.3.2 analysis using the metazoa_odb10 reference set ( *n* = 954) identified 79.9% of the expected gene set (single = 79.1%, duplicated = 0.7%).

## Metagenome report

**Figure 6.**
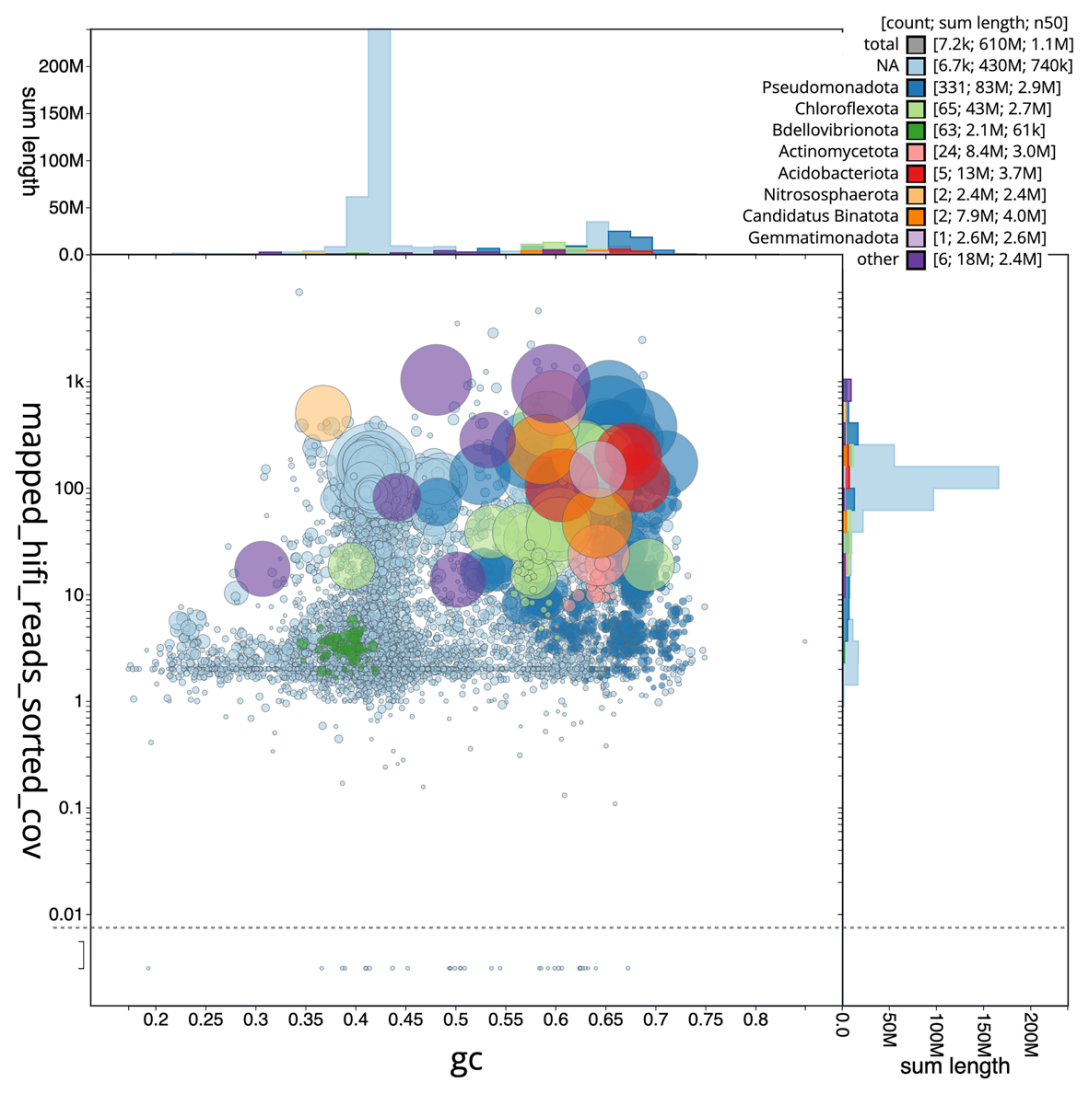
Blob plot of base coverage mapped against GC proportion for sequences in the metagenome of *Corticium candelabrum*. Binned metagenomes are coloured by phylum. Circles are sized in proportion to sequence length on a square root scale, ranging from 501 to 6,575,414. Histograms show the distribution of sequence length sum along each axis. An interactive version of this figure may be viewed here.

**Figure 7.**
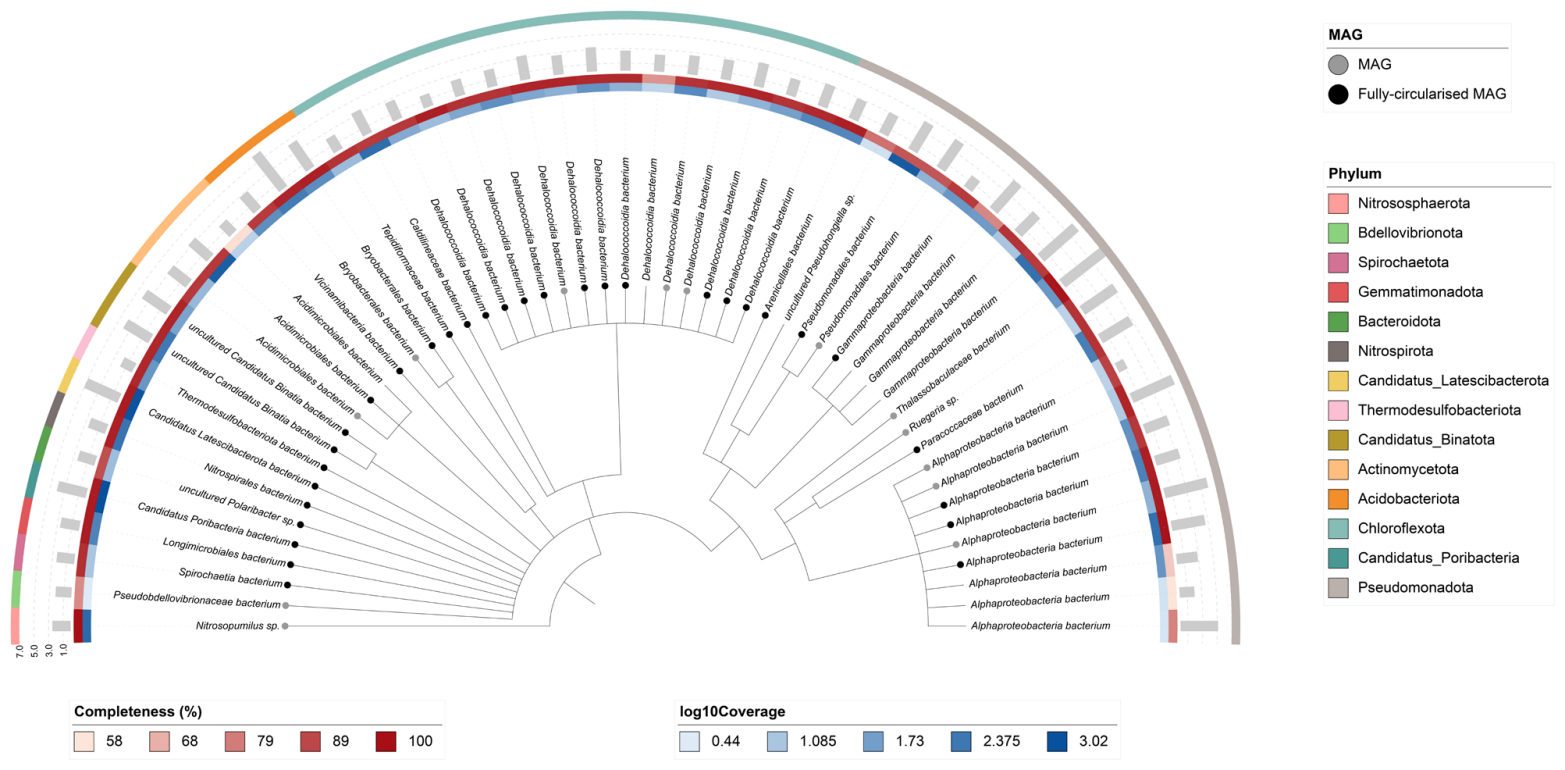
Cladogram showing the taxonomic placement of metagenome bins, constructed using NCBI taxonomic identifiers with *taxonomizr* and annotated in iTOL. Colours indicate phylum-level taxonomy. Additional tracks show sequencing coverage (log₁₀), genome size (Mbp), and completeness. Bins that meet the criteria for MAGs are marked with a grey circle; fully circularised MAGs are marked in black.

Fifty-three binned genomes were generated from the metagenome assembly ( Figure 6), of which 44 were classified as high-quality metagenome assembled genomes (MAGs) (see methods). The completeness values for these assemblies range from approximately 58% to 100% with contamination below 9%. A cladogram of the binned metagenomes is shown in Figure 7. (For details on binned genomes see https://doi.org/10.6084/m9.figshare.30017644.)

## Genome annotation report

The *Corticium candelabrum* genome assembly (GCA_963422355.1) was annotated at the European Bioinformatics Institute (EBI) using the Ensembl Genebuild pipeline. The resulting annotation includes 40,526 transcribed mRNAs from 26,198 protein-coding and 413 non-coding genes ( Table 2; https://beta.ensembl.org/species/9d1ccad7-a202-4858-9275-8224ad9515df). The average transcript length is 5,686.84, with an average of 1.52 coding transcripts per gene and 6.81 exons per transcript.

## Methods

### Sample acquisition

An adult *Corticium candelabrum* (specimen ID GHC0000185, ToLID ooCorCand1) was collected from Blanes, Girona, Spain (latitude 41.67333, longitude 2.80314) on 2021-02-01 by scuba diving. The specimen was collected and identified by Manuel Maldonado (CEAB-CSIC) and preserved by snap-freezing.

### Nucleic acid extraction

The workflow for high molecular weight (HMW) DNA extraction at the Wellcome Sanger Institute (WSI) Tree of Life Core Laboratory includes a sequence of procedures: sample preparation and homogenisation, DNA extraction, fragmentation and purification. Detailed protocols are available on protocols.io ( Denton *et al.*, 2023). The ooCorCand1 sample was prepared for DNA extraction by weighing and dissecting it on dry ice ( Jay *et al.*, 2023). Tissue was cryogenically disrupted using the Covaris cryoPREP ^®^ Automated Dry Pulverizer ( Narváez-Gómez *et al.*, 2023). HMW DNA was extracted using the Manual MagAttract v1 protocol ( Strickland *et al.*, 2023b). DNA was sheared into an average fragment size of 12–20 kb in a Megaruptor 3 system ( Todorovic *et al.*, 2023). Sheared DNA was purified by solid-phase reversible immobilisation, using AMPure PB beads to eliminate shorter fragments and concentrate the DNA ( Strickland *et al.*, 2023a). The concentration of the sheared and purified DNA was assessed using a Nanodrop spectrophotometer and Qubit Fluorometer using the Qubit dsDNA High Sensitivity Assay kit. Fragment size distribution was evaluated by running the sample on the FemtoPulse system.

RNA was also extracted from tissue of ooCorCand1 in the Tree of Life Laboratory at the WSI using the RNA Extraction: Automated MagMax™ *mir*Vana protocol ( do Amaral *et al.*, 2023). The RNA concentration was assessed using a Nanodrop spectrophotometer and a Qubit Fluorometer using the Qubit RNA Broad-Range Assay kit. Analysis of the integrity of the RNA was done using the Agilent RNA 6000 Pico Kit and Eukaryotic Total RNA assay.

### Sequencing

Pacific Biosciences HiFi circular consensus DNA sequencing libraries were constructed according to the manufacturers’ instructions. DNA sequencing was performed by the Scientific Operations core at the WSI on the Pacific Biosciences Sequel IIe instrument. Tissue from the ooCorCand1 sample was processed for Hi-C sequencing at the WSI Scientific Operations core, using the Arima-HiC v2 kit and sequenced on the Illumina NovaSeq 6000 instrument. Poly(A) RNA-Seq libraries were constructed using the NEB Ultra II RNA Library Prep kit, following the manufacturer’s instructions. RNA sequencing was performed on the Illumina NovaSeq X instrument.

### Genome assembly, curation and evaluation

***Assembly***

Prior to assembly of the PacBio HiFi reads, a database of *k*-mer counts ( *k* = 31) was generated from the filtered reads using FastK. GenomeScope2 ( Ranallo-Benavidez *et al.*, 2020) was used to analyse the *k*-mer frequency distributions, providing estimates of genome size, heterozygosity, and repeat content.

The HiFi reads were assembled using Hifiasm ( Cheng *et al.*, 2021) with the --primary option. Haplotypic duplications were identified and removed using purge_dups ( Guan *et al.*, 2020). The Hi-C reads were mapped to the primary contigs using bwa-mem2 ( Vasimuddin *et al.*, 2019). The contigs were further scaffolded using the provided Hi-C data ( Rao *et al.*, 2014) in YaHS ( Zhou *et al.*, 2023) using the --break option for handling potential misassemblies. The scaffolded assemblies were evaluated using Gfastats ( Formenti *et al.*, 2022), BUSCO ( Manni *et al.*, 2021) and MERQURY.FK ( Rhie *et al.*, 2020).

The mitochondrial genome was assembled using MitoHiFi ( Uliano-Silva *et al.*, 2023), which runs MitoFinder ( Allio *et al.*, 2020) and uses these annotations to select the final mitochondrial contig and to ensure the general quality of the sequence.

***Assembly curation***

The assembly was checked for contamination and corrected using the gEVAL system ( Chow *et al.*, 2016) as described previously ( Howe *et al.*, 2021). Manual curation was conducted primarily in PretextView ( Harry, 2022) and HiGlass ( Kerpedjiev *et al.*, 2018). Any identified contamination, missed joins, and mis-joins were amended, and duplicate sequences were tagged and removed. The curation process is documented at https://gitlab.com/wtsi-grit/rapid-curation.

***Assembly quality assessment***

A Hi-C contact map was produced for the final version of the assembly. The Hi-C reads were aligned using bwa-mem2 ( Vasimuddin *et al.*, 2019) and the alignment files were combined using SAMtools ( Danecek *et al.*, 2021). The Hi-C alignments were converted into a contact map using BEDTools ( Quinlan & Hall, 2010) and the Cooler tool suite ( Abdennur & Mirny, 2020). The contact map is visualised in HiGlass ( Kerpedjiev *et al.*, 2018). The Merqury.FK tool ( Rhie *et al.*, 2020), run in a Singularity container ( Kurtzer *et al.*, 2017), was used to evaluate the assembly QV scores. The genome was also analysed within the BlobToolKit environment ( Challis *et al.*, 2020) and BUSCO scores ( Manni *et al.*, 2021) were calculated. Table 4 contains a list of relevant software tool versions and sources.

**Table 4.** Software tools: versions and sources.

Software tool	Version	Source
BEDTools	2.30.0	https://github.com/arq5x/bedtools2
bin3C	0.3.3	https://github.com/cerebis/bin3C
BLAST	2.14.0	ftp://ftp.ncbi.nlm.nih.gov/blast/executables/blast+/
BlobToolKit	4.2.1	https://github.com/blobtoolkit/blobtoolkit
BUSCO	5.3.2	https://gitlab.com/ezlab/busco
bwa-mem2	2.2.1	https://github.com/bwa-mem2/bwa-mem2
CheckM	1.2.1	https://github.com/Ecogenomics/CheckM
Cooler	0.8.11	https://github.com/open2c/cooler
DIAMOND	2.0.15	https://github.com/bbuchfink/diamond
dRep	3.4.0	https://github.com/MrOlm/drep
fasta_windows	0.2.4	https://github.com/tolkit/fasta_windows
FastK	1.1	https://github.com/thegenemyers/FASTK
gEVAL	N/A	https://geval.org.uk/
Gfastats	1.3.6	https://github.com/vgl-hub/gfastats
GoaT CLI	0.2.5	https://github.com/genomehubs/goat-cli
GTDB-TK	2.3.2	https://github.com/Ecogenomics/GTDBTk
Hifiasm	0.16.1	https://github.com/chhylp123/hifiasm
HiGlass	1.13.4	https://github.com/higlass/higlass
MAGScoT	1.0.0	https://github.com/ikmb/MAGScoT
MaxBin	2.7	https://sourceforge.net/projects/maxbin/
MerquryFK	1.1	https://github.com/thegenemyers/MERQURY.FK
MetaBat2	2.15-15-gd6ea400	https://bitbucket.org/berkeleylab/metabat/src/master/
metaMDBG	Pre-release	https://github.com/GaetanBenoitDev/metaMDBG
MetaTOR	-	https://github.com/koszullab/metaTOR
Minimap2	2.24-r1122	https://github.com/lh3/minimap2
MitoHiFi	2	https://github.com/marcelauliano/MitoHiFi
MultiQC	1.14, 1.17, and 1.18	https://github.com/MultiQC/MultiQC
PretextView	0.2.5	https://github.com/sanger-tol/PretextView
PROKKA	1.14.5	https://github.com/vdejager/prokka
purge_dups	1.2.3	https://github.com/dfguan/purge_dups
samtools	1.15.1	https://github.com/samtools/samtools
Seqtk	1.3	https://github.com/lh3/seqtk
Singularity	3.9.0	https://github.com/sylabs/singularity
YaHS	1.1a.2	https://github.com/c-zhou/yahs

***Taxonomic verification***

There are no published sequences of the *Corticium candelabrum* holotype (LMJG 15353/0) or other type material. For molecular taxonomic verification CO1 (Folmer) and 28S rDNA (C-Region) barcoding markers of the present sample were compared against sequences currently published in NCBI Genbank as *C. candelabrum* using MAFFT ( Katoh & Standley, 2013). The CO1 marker of present sample is identical to *C. candelabrum* JX999073 of Riesgo *et al.* (2014). However, alignments displayed only 94.5 % similarity (CO1) and 78% similarity (28S) to a *C. candelabrum* specimen published by Gazave *et al*. (2010) (HQ269363 and HM118553 respectively). As the latter specimen was collected in a cave environment (Coral Cave, Marseilles, France, see Gazave *et al.* (2010), which is not mentioned in Schmidt's original description of *C. candelabrum* (1862), we assume it being a different species.

### Genome annotation

The Ensembl Genebuild annotation system ( Aken *et al.*, 2016) was used to generate annotation for the *Corticium candelabrum* assembly (GCA_963422355.1) in Ensembl Rapid Release at the EBI. Annotation was created primarily through alignment of transcriptomic data to the genome, with gap filling via protein-to-genome alignments of a select set of proteins from UniProt ( UniProt Consortium, 2019).

### Metagenome assembly

The metagenome assembly was generated using MetaMDBG ( Benoit *et al.*, 2024) and binned using MetaBAT2 ( Kang *et al.*, 2019), MaxBin ( Wu *et al.*, 2014), bin3C ( DeMaere & Darling, 2019), and MetaTOR. The resulting bin sets of each binning algorithm were optimised and refined using MAGScoT ( Rühlemann *et al.*, 2022). PROKKA ( Seemann, 2014) was used to identify tRNAs and rRNAs in each bin, CheckM ( Parks *et al.*, 2015) (checkM_DB release 2015-01-16) was used to assess bin completeness/contamination, and GTDB-TK ( Chaumeil *et al.*, 2022) (GTDB release 214) was used to taxonomically classify bins. Taxonomic replicate bins were identified using dRep ( Olm *et al.*, 2017) with default settings (95% ANI threshold). The final bin set was filtered for bacteria and archaea. All bins were assessed for quality and categorised as metagenome-assembled genomes (MAGs) if they met the following criteria: contamination ≤ 5%, presence of 5S, 16S, and 23S rRNA genes, at least 18 unique tRNAs, and either ≥ 90% completeness or ≥ 50% completeness with fully circularised chromosomes. Bins that did not meet these thresholds, or were identified as taxonomic replicates of MAGs, were retained as ‘binned metagenomes’ provided they had ≥ 50% completeness and ≤ 10% contamination. A cladogram based on NCBI taxonomic assignments was generated using the ‘taxonomizr’ package in R. The tree was visualised and annotated using iTOL ( Letunic & Bork, 2024). Software tool versions and sources are given in Table 4.

### Wellcome Sanger Institute – Legal and Governance

The materials that have contributed to this genome note have been supplied by a Tree of Life collaborator. The Wellcome Sanger Institute employs a process whereby due diligence is carried out proportionate to the nature of the materials themselves, and the circumstances under which they have been/are to be collected and provided for use. The purpose of this is to address and mitigate any potential legal and/or ethical implications of receipt and use of the materials as part of the research project, and to ensure that in doing so we align with best practice wherever possible. The overarching areas of consideration are:

•   Ethical review of provenance and sourcing of the material

•   Legality of collection, transfer and use (national and international)

Each transfer of samples is undertaken according to a Research Collaboration Agreement or Material Transfer Agreement entered into by the Tree of Life collaborator, Genome Research Limited (operating as the Wellcome Sanger Institute) and in some circumstances other Tree of Life collaborators.

## Data Availability

European Nucleotide Archive: Corticium candelabrum. Accession number PRJEB64714; https://identifiers.org/ena.embl/PRJEB64714. The genome sequence is released openly for reuse. The *Corticium candelabrum* genome sequencing initiative is part of the Aquatic Symbiosis Genomics (ASG) project ( https://www.ebi.ac.uk/ena/browser/view/PRJEB43743). All raw sequence data and the assembly have been deposited in INSDC databases. Raw data and assembly accession identifiers are reported in Table 1 and Table 2.

## Author information

Contributors are listed at the following links:

- Wellcome Sanger Institute Tree of Life Management, Samples and Laboratory team
- Wellcome Sanger Institute Scientific Operations – Sequencing Operations
- Wellcome Sanger Institute Tree of Life Core Informatics team
- Tree of Life Core Informatics collective
- European Bioinformatics Institute ASG Data Portal team
- Wellcome Sanger Institute/Aquatic Symbiosis Genomics Project Leadership
