# Supplementary material for: The chromosomal genome sequence of the sponge, Corticium candelabrum Schmidt, 1862 and its associated microbial metagenome sequences
Source: Wellcome Open Res. 2025 Oct 3;10:535. [Version 1] doi: 10.12688/wellcomeopenres.24923.1 (PMC12586988; doi:10.12688/wellcomeopenres.24923.1)
Supplement: Peer review history

# Reviewer report: Reviewer response for version 1

**DOI:** 10.21956/wellcomeopenres.27453.r136086

**Published:** 2025-11-04

**Authors:** Richard R Copley

**Affiliations:** 1 Laboratoire de Biologie du Developpement de Villefranche-sur-Mer, Villefranche-sur-Mer, Provence-Alpes-Côte d'Azur, France

The article presents a chromosome level assembly of the homoscleromorph sponge *Corticium candelabrum*. The genome is of high quality and assembled into 22 pseudo-chromosomes. A set of predicted proteins produced using the ensembl gene build pipeline are also made available. By the standards of other animals these have a relatively low BUSCO score at 79.9%, but this appears comparable to reported values in other sponges. I think as a homoscleromorph this is one of the more interesting species to have had its genome reported recently, and given the constraints of Tree of Life publications, the authors do a reasonable job of presenting why that is the case. Regarding spicule evolution, 2 unpublished works of the first author are mentioned, but not, for instance.

Are sufficient details of methods and materials provided to allow replication by others?

Yes

Is the rationale for creating the dataset(s) clearly described?

Yes

Are the datasets clearly presented in a useable and accessible format?

Yes

Are the protocols appropriate and is the work technically sound?

Yes

Reviewer Expertise:

protein and genome research

I confirm that I have read this submission and believe that I have an appropriate level of expertise to confirm that it is of an acceptable scientific standard.

## References

[1] : Independent origins of spicules reconcile palaeontological and molecular evidence of the evolutionary history of sponges. *bioRxiv* .2024; 10.1101/2024.06.24.600355 10.1101/2024.06.24.600355 PMC1277806341499514

# Reviewer report: Reviewer response for version 1

**DOI:** 10.21956/wellcomeopenres.27453.r135128

**Published:** 2025-11-04

**Authors:** Jayan Duminda M Senevirathna

**Affiliations:** 1 Uva Wellassa University, Badulla, Uva Province, Sri Lanka

The article is well-written and easy to follow. This genome will be a valuable reference for phylogenomic or conservation studies.

 The following are suggestions for further improvements.

 The abbreviation "COI" is the correct form for the cytochrome c oxidase subunit I gene. Please check and correct the Taxonomic verification section. Always use 28S rDNA in all places.

Are sufficient details of methods and materials provided to allow replication by others?

Yes

Is the rationale for creating the dataset(s) clearly described?

Yes

Are the datasets clearly presented in a useable and accessible format?

Yes

Are the protocols appropriate and is the work technically sound?

Yes

Reviewer Expertise:

Aquatic Biotechnology and Genetics

I confirm that I have read this submission and believe that I have an appropriate level of expertise to confirm that it is of an acceptable scientific standard.

# Reviewer report: Reviewer response for version 1

**DOI:** 10.21956/wellcomeopenres.27453.r135127

**Published:** 2025-10-25

**Authors:** Estelle Proux-Wéra

**Affiliations:** 1 Stockholm University, stockholm, Sweden

In this article the authors assembled the genome of the sponge *Corticium candelabrum *and its mitochondrial genome, and studied the metagenome of the specimen.

 The data (raw and final assemblies) is findable and downloadable.

 The methods used for assembling the genome, mitochondrial genome, and identifying the metagenome are modern. The name of the tools and their versions are provided, however we dont know about any specific parameters used, or if some sanger-tol pipelines were used.

 The results looks similar to other sponge genomes in term of genome size and metagenome content. The majority of the assembly is in 22 long chromosomal pseudomolecules. The assembly metrics look good.

Are sufficient details of methods and materials provided to allow replication by others?

Partly

Is the rationale for creating the dataset(s) clearly described?

Yes

Are the datasets clearly presented in a useable and accessible format?

Yes

Are the protocols appropriate and is the work technically sound?

Yes

Reviewer Expertise:

Bioinformatics, genome assembly

I confirm that I have read this submission and believe that I have an appropriate level of expertise to confirm that it is of an acceptable scientific standard.
